# Supplementary material for: Whole-genome comparative analysis at the lineage/sublineage level discloses relationships between Mycobacterium tuberculosis genotype and clinical phenotype
Source: PeerJ. 2021 Sep 8;9:e12128. doi: 10.7717/peerj.12128 (PMC8434806; doi:10.7717/peerj.12128)
Supplement: Supplemental Information 1 [file peerj-09-12128-s001.docx]

| **Supplemental Table 1**. Raw genome data strains. | | | | | |  |
| --- | --- | --- | --- | --- | --- | --- |
| No. | Strain | Site of infection | Disease type | SRA | Country |  |
| 1 | DCTB0011 | EPTB | EPTB | SRR998860 | Russia |  |
| 2 | Index 78-34 | Central nervous system | EPTB | SRR6480455 | Indonesia |  |
| 3 | ETB0119 | Bone and joints | EPTB | SRR6257091 | Russia |  |
| 4 | 48-10477 | Central nervous system | EPTB | SRR5709859 | Thailand |  |
| 5 | Index 4-42 | Central nervous system | EPTB | SRR6480537 | Indonesia |  |
| 6 | Index 28-66 | Central nervous system | EPTB | SRR6480585 | Indonesia |  |
| 7 | Index 60-88 | Central nervous system | EPTB | SRR6480481 | Indonesia |  |
| 8 | Index 66-88 | Central nervous system | EPTB | SRR6480501 | Indonesia |  |
| 9 | TB0002 | Bone and joints | EPTB | SRR6256980 | Russia |  |
| 10 | TB0010 | Bone and joints | EPTB | SRR6256978 | Russia |  |
| 11 | L-92954 | Central nervous system | EPTB | SRR5709831 | Thailand |  |
| 12 | Index 4-66 | Central nervous system | EPTB | SRR6480445 | Indonesia |  |
| 13 | DS-21277 | Central nervous system | EPTB | SRR5709931 | Thailand |  |
| 14 | TB0068 | Bone and joints | EPTB | SRR6257006 | Russia |  |
| 15 | CSF-3367 | Central nervous system | EPTB | SRR5709804 | Thailand |  |
| 16 | Index 60-19 | Central nervous system | EPTB | SRR6480609 | Indonesia |  |
| 17 | 4-12339 | Central nervous system | EPTB | SRR5709998 | Thailand |  |
| 18 | TB0059 | Bone and joints | EPTB | SRR6256986 | Russia |  |
| 19 | 43-12700 | Central nervous system | EPTB | SRR5710006 | Thailand |  |
| 20 | Index 4-88 | Central nervous system | EPTB | SRR6480528 | Indonesia |  |
| 21 | 42-19187 | Central nervous system | EPTB | SRR5709980 | Thailand |  |
| 22 | DCTB0012 | EPTB | EPTB | SRR998861 | Russia |  |
| 23 | Index 45-88 | Central nervous system | EPTB | SRR6480525 | Indonesia |  |
| 24 | Index 18-66 | Central nervous system | EPTB | SRR6480582 | Indonesia |  |
| 25 | TB0079 | Bone and joints | EPTB | SRR6257013 | Russia |  |
| 26 | Mtb-manila-23 | Genitourinary system | EPTB | SRR1510058 | Canada |  |
| 27 | CSF3943 | Central nervous system | EPTB | SRR5709999 | Thailand |  |
| 28 | CSF-4438 | Central nervous system | EPTB | SRR5710029 | Thailand |  |
| 29 | CSF-2894 | Central nervous system | EPTB | SRR5709977 | Thailand |  |
| 30 | 46-18357 | Central nervous system | EPTB | SRR5709997 | Thailand |  |
| 31 | 46-19260 | Central nervous system | EPTB | SRR5709971 | Thailand |  |
| 32 | 43-19186 | Central nervous system | EPTB | SRR5710001 | Thailand |  |
| 33 | 47-17522 | Central nervous system | EPTB | SRR5709742 | Thailand |  |
| 34 | Index 55-19 | Central nervous system | EPTB | SRR6480620 | Indonesia |  |
| 35 | DCTB0004 | EPTB | EPTB | SRR998140 | Russia |  |
| 36 | TB0036 | Bone and joints | EPTB | SRR6257086 | Russia |  |
| 37 | CSF-4129 | Central nervous system | EPTB | SRR5709834 | Thailand |  |
| 38 | Index 30-88 | Central nervous system | EPTB | SRR6480394 | Indonesia |  |
| 39 | Index 73-19 | Central nervous system | EPTB | SRR6480381 | Indonesia |  |
| 40 | Index 12-88 | Central nervous system | EPTB | SRR6480407 | Indonesia |  |
| 41 | DCTB0006 | EPTB | EPTB | SRR998856 | Russia |  |
| 42 | Index 45-42 | Central nervous system | EPTB | SRR6480500 | Indonesia |  |
| 43 | Index 21-42 | Central nervous system | EPTB | SRR6480416 | Indonesia |  |
| 44 | Index 40-34 | Central nervous system | EPTB | SRR6480472 | Thailand |  |
| 45 | DKC2-0499 | Lymph node | EPTB | ERR2229374 | Denmark |  |
| 46 | CSF-4374 | Central nervous system | EPTB | SRR5709861 | Thailand |  |
| 47 | 46-10698 | Central nervous system | EPTB | SRR5709739 | Thailand |  |
| 48 | CSF4676 | Central nervous system | EPTB | SRR5709975 | Thailand |  |
| 49 | CSF-3518 | Central nervous system | EPTB | SRR5709788 | Thailand |  |
| 50 | Index 24-66 | Central nervous system | EPTB | SRR6480584 | Indonesia |  |
| 51 | TB0092 | Bone and joints | EPTB | SRR6257024 | Russia |  |
| 52 | Index 30-19 | Central nervous system | EPTB | SRR6480624 | Indonesia |  |
| 53 | Index 21-34 | Central nervous system | EPTB | SRR6480435 | Indonesia |  |
| 54 | Index 6-101 | Central nervous system | EPTB | SRR6480557 | Indonesia |  |
| 55 | TB0039 | Bone and joints | EPTB | SRR6257085 | Russia |  |
| 56 | PGI_IOB_EPTB5 | Lymph node | EPTB | SRR5125077 | India |  |
| 57 | CSF-3382 | Central nervous system | EPTB | SRR5709783 | Thailand |  |
| 58 | Index 73-88 | Central nervous system | EPTB | SRR6480477 | Indonesia |  |
| 59 | 43-08441 | Central nervous system | EPTB | SRR5709985 | Thailand |  |
| 60 | Index 6-19 | Central nervous system | EPTB | SRR6480373 | Indonesia |  |
| 61 | Index 34-88 | Central nervous system | EPTB | SRR6480521 | Indonesia |  |
| 62 | Index 73-34 | Central nervous system | EPTB | SRR6480457 | Indonesia |  |
| 63 | CSF-4049 | Central nervous system | EPTB | SRR5709857 | Thailand |  |
| 64 | 43-12683 | Central nervous system | EPTB | SRR5709973 | Thailand |  |
| 65 | CSF-4229 | Central nervous system | EPTB | SRR5709836 | Thailand |  |
| 66 | DKC2-0T97 | Genitourinary system | EPTB | ERR1950087 | Denmark |  |
| 67 | CSF-3541 | Central nervous system | EPTB | SRR5709779 | Thailand |  |
| 68 | Index 61-19 | Central nervous system | EPTB | SRR6480626 | Indonesia |  |
| 69 | L-78532 | Central nervous system | EPTB | SRR5709800 | Thailand |  |
| 70 | CSF-3538 | Central nervous system | EPTB | SRR5709789 | Thailand |  |
| 71 | Index 8-34 | Central nervous system | EPTB | SRR6480454 | Indonesia |  |
| 72 | Index 28-88 | Central nervous system | EPTB | SRR6480398 | Indonesia |  |
| 73 | DKC2-0843 | Genitourinary system | EPTB | ERR2229774 | Denmark |  |
| 74 | Index 37-19 | Central nervous system | EPTB | SRR6480631 | Indonesia |  |
| 75 | Index 12-20 | Central nervous system | EPTB | SRR6480452 | Indonesia |  |
| 76 | 43-15038 | Central nervous system | EPTB | SRR5710003 | Thailand |  |
| 77 | CSF-3681 | Central nervous system | EPTB | SRR5709746 | Thailand |  |
| 78 | 15-1149-0143 | Genitourinary system | EPTB | SRR6367399 | Australia |  |
| 79 | 47-5556 | Central nervous system | EPTB | SRR5709784 | Thailand |  |
| 80 | TB0055 | Bone and joints | EPTB | SRR6256990 | Russia |  |
| 81 | PGI_IOB_EPTB1 | Central nervous system | EPTB | SRR5125076 | India |  |
| 82 | DKC2-0244 | Genitourinary system | EPTB | ERR2229064 | Denmark |  |
| 83 | Index 34-34 | Central nervous system | EPTB | SRR6480424 | Indonesia |  |
| 84 | Index 45-19 | Central nervous system | EPTB | SRR6480606 | Indonesia |  |
| 85 | 43-13037 | Central nervous system | EPTB | SRR5710005 | Thailand |  |
| 86 | Index 12-66 | Central nervous system | EPTB | SRR6480376 | Indonesia |  |
| 87 | Index 15-20 | Central nervous system | EPTB | SRR6480363 | Indonesia |  |
| 88 | 17-3391-0148 | Genitourinary system | EPTB | SRR6339646 | Australia |  |
| 89 | Index 78-25 | Central nervous system | EPTB | SRR6480612 | Indonesia |  |
| 90 | Index 32-19 | Central nervous system | EPTB | SRR6480385 | Indonesia |  |
| 91 | TB0006 | Bone and joints | EPTB | SRR6256975 | Russia |  |
| 92 | PGI_IOB_EPTB2 | Lymph node | EPTB | SRR5125078 | India |  |
| 93 | Index 34-20 | Central nervous system | EPTB | SRR6480370 | Indonesia |  |
| 94 | DKC2-0246 | Bone and joints | EPTB | ERR2229066 | Denmark |  |
| 95 | CSF-4056 | Central nervous system | EPTB | SRR5709856 | Thailand |  |
| 96 | Index 42-34 | Central nervous system | EPTB | SRR6480471 | Indonesia |  |
| 97 | 43-11791 | Central nervous system | EPTB | SRR5709974 | Thailand |  |
| 98 | Index 8-101 | Central nervous system | EPTB | SRR6480555 | Indonesia |  |
| 99 | Index 6-88 | Central nervous system | EPTB | SRR6480508 | Indonesia |  |
| 100 | TB0008 | Bone and joints | EPTB | SRR6256976 | Russia |  |
| 101 | TB0048 | Bone and joints | EPTB | SRR6257109 | Russia |  |
| 102 | Index 12-19 | Central nervous system | EPTB | SRR6480339 | Indonesia |  |
| 103 | Index 8-19 | Central nervous system | EPTB | SRR6480608 | Indonesia |  |
| 104 | Index 55-34 | Central nervous system | EPTB | SRR6480469 | Indonesia |  |
| 105 | 48-10563 | Central nervous system | EPTB | SRR5709853 | Thailand |  |
| 106 | PGI_IOB_EPTB4 | Lymph node | EPTB | SRR5125075 | India |  |
| 107 | Index 8-20 | Central nervous system | EPTB | SRR6480483 | Indonesia |  |
| 108 | Index 61-88 | Central nervous system | EPTB | SRR6480482 | Indonesia |  |
| 109 | 43-01910 | Central nervous system | EPTB | SRR5709979 | Thailand |  |
| 110 | Mtb-manila-1 | Lymph node | EPTB | SRR1510036 | Canada |  |
| 111 | Index 66-20 | Central nervous system | EPTB | SRR6480487 | Indonesia |  |
| 112 | TB0157 | Bone and joints | EPTB | SRR6257056 | Russia |  |
| 113 | Index 21-66 | Central nervous system | EPTB | SRR6480583 | Indonesia |  |
| 114 | Index 18-20 | Central nervous system | EPTB | SRR6480364 | Indonesia |  |
| 115 | Index 30-66 | Central nervous system | EPTB | SRR6480397 | Indonesia |  |
| 116 | TB0040 | Bone and joints | EPTB | SRR6257082 | Russia |  |
| 117 | 48-10231 | Central nervous system | EPTB | SRR5709852 | Thailand |  |
| 118 | CSF-4666 | Central nervous system | EPTB | SRR5709745 | Thailand |  |
| 119 | CSF-3419 | Central nervous system | EPTB | SRR5709995 | Thailand |  |
| 120 | Index 55-20 | Central nervous system | EPTB | SRR6480491 | Indonesia |  |
| 121 | Index 45-20 | Central nervous system | EPTB | SRR6480492 | Indonesia |  |
| 122 | CSF-4549 | Central nervous system | EPTB | SRR5709790 | Thailand |  |
| 123 | TB0080 | Bone and joints | EPTB | SRR6257027 | Russia |  |
| 124 | CSF-4261 | Central nervous system | EPTB | SRR5709830 | Thailand |  |
| 125 | TB0085 | Bone and joints | EPTB | SRR6257031 | Russia |  |
| 126 | Index 21-20 | Central nervous system | EPTB | SRR6480365 | Indonesia |  |
| 127 | TB0053 | Bone and joints | EPTB | SRR6257100 | Russia |  |
| 128 | Index 40-19 | Central nervous system | EPTB | SRR6480627 | Indonesia |  |
| 129 | 43-11467 | Central nervous system | EPTB | SRR5709983 | Thailand |  |
| 130 | TB0042 | Bone and joints | EPTB | SRR6257080 | Russia |  |
| 131 | Index 15-66 | Central nervous system | EPTB | SRR6480378 | Indonesia |  |
| 132 | TB0054 | Bone and joints | EPTB | SRR6256989 | Russia |  |
| 133 | Index 24-19 | Central nervous system | EPTB | SRR6480623 | Indonesia |  |
| 134 | Mtb40 | Lymph node | EPTB | SRR5817466 | Australia |  |
| 135 | Index 12-34 | Central nervous system | EPTB | SRR6480432 | Indonesia |  |
| 136 | CSF-3542 | Central nervous system | EPTB | SRR5709778 | Thailand |  |
| 137 | CSF-4620 | Central nervous system | EPTB | SRR5709832 | Thailand |  |
| 138 | Index 32-34 | Central nervous system | EPTB | SRR6480439 | Indonesia |  |
| 139 | CSF-4396 | Central nervous system | EPTB | SRR5709828 | Thailand |  |
| 140 | TB0064 | Bone and joints | EPTB | SRR6256993 | Russia |  |
| 141 | Index 4-34 | Central nervous system | EPTB | SRR6480473 | Indonesia |  |
| 142 | Index 18-19 | Central nervous system | EPTB | SRR6480565 | Indonesia |  |
| 143 | TB0088 | Bone and joints | EPTB | SRR6257030 | Russia |  |
| 144 | Index 75-34 | Central nervous system | EPTB | SRR6480456 | Indonesia |  |
| 145 | Index 6-20 | Central nervous system | EPTB | SRR6480490 | Indonesia |  |
| 146 | Index 55-88 | Central nervous system | EPTB | SRR6480430 | Indonesia |  |
| 147 | Index 8-88 | Central nervous system | EPTB | SRR6480480 | Indonesia |  |
| 148 | TB0044 | Bone and joints | EPTB | SRR6257105 | Russia |  |
| 149 | Index 78-88 | Central nervous system | EPTB | SRR6480479 | Indonesia |  |
| 150 | Index 37-42 | Central nervous system | EPTB | SRR6480543 | Indonesia |  |
| 151 | ETB0090 | Bone and joints | EPTB | SRR6257034 | Russia |  |
| 152 | DCTB0009 | EPTB | EPTB | SRR998858 | Russia |  |
| 153 | Index 69-88 | Central nervous system | EPTB | SRR6480605 | Indonesia |  |
| 154 | Index 15-34 | Central nervous system | EPTB | SRR6480433 | Indonesia |  |
| 155 | TB0113 | Bone and joints | EPTB | SRR6257097 | Russia |  |
| 156 | 42-20464 | Central nervous system | EPTB | SRR5709741 | Thailand |  |
| 157 | Index 78-19 | Central nervous system | EPTB | SRR6480566 | Indonesia |  |
| 158 | Index 40-20 | Central nervous system | EPTB | SRR6480371 | Indonesia |  |
| 159 | DCTB0008 | EPTB | EPTB | SRR998857 | Russia |  |
| 160 | CSF-3922 | Central nervous system | EPTB | SRR5709854 | Thailand |  |
| 161 | CSF-4202 | Central nervous system | EPTB | SRR5709835 | Thailand |  |
| 162 | Index 4-101 | Central nervous system | EPTB | SRR6480562 | Indonesia |  |
| 163 | Index 60-20 | Central nervous system | EPTB | SRR6480489 | Indonesia |  |
| 164 | TB0012 | Bone and joints | EPTB | SRR6256984 | Russia |  |
| 165 | CSF-4105 | Central nervous system | EPTB | SRR5709833 | Thailand |  |
| 166 | CSF-4069 | Central nervous system | EPTB | SRR5709851 | Thailand |  |
| 167 | Mtb-manila-27 | Lymph node | EPTB | SRR1510062 | Canada |  |
| 168 | Index 42-40 | Central nervous system | EPTB | SRR6480372 | Indonesia |  |
| 169 | CSF-1449 | Central nervous system | EPTB | SRR5709972 | Thailand |  |
| 170 | Index 6-34 | Central nervous system | EPTB | SRR6480468 | Indonesia |  |
| 171 | Index 4-19 | Central nervous system | EPTB | SRR6480632 | Indonesia |  |
| 172 | CSF-4480 | Central nervous system | EPTB | SRR5709838 | Thailand |  |
| 173 | Mtb-33 | Lymph node | EPTB | SRR5817467 | Australia |  |
| 174 | TB0058 | Bone and joints | EPTB | SRR6256985 | Russia |  |
| 175 | TB0086 | Bone and joints | EPTB | SRR6257032 | Russia |  |
| 176 | TB0057 | Bone and joints | EPTB | SRR6256992 | Russia |  |
| 177 | CSF-3384 | Central nervous system | EPTB | SRR5709786 | Thailand |  |
| 178 | Index 21-88 | Central nervous system | EPTB | SRR6480395 | Indonesia |  |
| 179 | TB0011 | Bone and joints | EPTB | SRR6256983 | Russia |  |
| 180 | 43-05286 | Central nervous system | EPTB | SRR5709986 | Thailand |  |
| 181 | 47-10904 | Central nervous system | EPTB | SRR5709785 | Thailand |  |
| 182 | Index 75-88 | Central nervous system | EPTB | SRR6480478 | Indonesia |  |
| 183 | TB0037 | Bone and joints | EPTB | SRR6257087 | Russia |  |
| 184 | Index 32-88 | Central nervous system | EPTB | SRR6480522 | Indonesia |  |
| 185 | TB0047 | Bone and joints | EPTB | SRR6257102 | Russia |  |
| 186 | 43-13718 | Central nervous system | EPTB | SRR5710004 | Thailand |  |
| 187 | Index 61-34 | Central nervous system | EPTB | SRR6480466 | Indonesia |  |
| 188 | 14-14745 | Central nervous system | EPTB | SRR5709740 | Thailand |  |
| 189 | Index 42-88 | Central nervous system | EPTB | SRR6480526 | Indonesia |  |
| 190 | Index 30-42 | Central nervous system | EPTB | SRR6480414 | Indonesia |  |
| 191 | Index 28-20 | Central nervous system | EPTB | SRR6480367 | Indonesia |  |
| 192 | Index 66-34 | Central nervous system | EPTB | SRR6480476 | Indonesia |  |
| 193 | Index 61-20 | Central nervous system | EPTB | SRR6480488 | Indonesia |  |
| 194 | 48-4979 | Central nervous system | EPTB | SRR5709855 | Thailand |  |
| 195 | Mtb-manila-14 | Central nervous system | EPTB | SRR1510049 | Canada |  |
| 196 | Index 15-88 | Central nervous system | EPTB | SRR6480404 | Indonesia |  |
| 197 | CSF-4115 | Central nervous system | EPTB | SRR5709850 | Thailand |  |
| 198 | Mtb-manila-22 | Bone and joints | EPTB | SRR1510057 | Canada |  |
| 199 | TB0038 | Bone and joints | EPTB | SRR6257084 | Russia |  |
| 200 | CSF-4014 | Central nervous system | EPTB | SRR5709858 | Thailand |  |
| 201 | TB0081 | Bone and joints | EPTB | SRR6257028 | Russia |  |
| 202 | Index 66-19 | Central nervous system | EPTB | SRR6480331 | Indonesia |  |
| 203 | CSF-3711 | Central nervous system | EPTB | SRR5709747 | Thailand |  |
| 204 | DCTB0005 | EPTB | EPTB | SRR998855 | Russia |  |
| 205 | Index 28-34 | Central nervous system | EPTB | SRR6480437 | Indonesia |  |
| 206 | Index 24-20 | Central nervous system | EPTB | SRR6480366 | Indonesia |  |
| 207 | CSF2498 | Central nervous system | EPTB | SRR5709976 | Thailand |  |
| 208 | CSF-3346 | Central nervous system | EPTB | SRR5709791 | Thailand |  |
| 209 | CSF-3718 | Central nervous system | EPTB | SRR5709744 | Thailand |  |
| 210 | DKC2-0T40 | Lymph node | EPTB | ERR2229808 | Denmark |  |
| 211 | 72-125842 | Central nervous system | EPTB | SRR5710000 | Thailand |  |
| 212 | Index 24-88 | Central nervous system | EPTB | SRR6480396 | Indonesia |  |
| 213 | Index 34-19 | Central nervous system | EPTB | SRR6480619 | Indonesia |  |
| 214 | Index 69-19 | Central nervous system | EPTB | SRR6480333 | Indonesia |  |
| 215 | TB0051 | Bone and joints | EPTB | SRR6257106 | Russia |  |
| 216 | Index 12-42 | Central nervous system | EPTB | SRR6480460 | Indonesia |  |
| 217 | Index 78-20 | Central nervous system | EPTB | SRR6480484 | Indonesia |  |
| 218 | PGI_IOB_EPTB3 | Lymph node | EPTB | SRR5125074 | India |  |
| 219 | Index 40-88 | Central nervous system | EPTB | SRR6480527 | Indonesia |  |
| 220 | TB0083 | Bone and joints | EPTB | SRR6257025 | Russia |  |
| 221 | CSF-4248 | Central nervous system | EPTB | SRR5709829 | Thailand |  |
| 222 | Index 32-20 | Central nervous system | EPTB | SRR6480369 | Indonesia |  |
| 223 | Index 75-20 | Central nervous system | EPTB | SRR6480485 | Indonesia |  |
| 224 | Index 18-34 | Central nervous system | EPTB | SRR6480434 | Indonesia |  |
| 225 | TB0043 | Bone and joints | EPTB | SRR6257081 | Russia |  |
| 226 | 4317298 | Central nervous system | EPTB | SRR5710002 | Thailand |  |
| 227 | Index 6-66 | Central nervous system | EPTB | SRR6480448 | Indonesia |  |
| 228 | CSF-3053 | Central nervous system | EPTB | SRR5709978 | Thailand |  |
| 229 | Index 69-34 | Central nervous system | EPTB | SRR6480475 | Indonesia |  |
| 230 | 43-11359 | Central nervous system | EPTB | SRR5709984 | Thailand |  |
| 231 | Index 73-20 | Central nervous system | EPTB | SRR6480486 | Indonesia |  |
| 232 | DCTB0002 | EPTB | EPTB | SRR993140 | Russia |  |
| 233 | Mtb-manila-25 | Genitourinary system | EPTB | SRR1510060 | Canada |  |
| 234 | CSF-3520 | Central nervous system | EPTB | SRR5709787 | Thailand |  |
| 235 | Index 8-66 | Central nervous system | EPTB | SRR6480406 | Indonesia |  |
| 236 | Index 42-19 | Central nervous system | EPTB | SRR6480629 | Indonesia |  |
| 237 | CSF-4409 | Central nervous system | EPTB | SRR5709939 | Thailand |  |
| 238 | TB0050 | Bone and joints | EPTB | SRR6257107 | Russia |  |
| 239 | Index 18-88 | Central nervous system | EPTB | SRR6480405 | Indonesia |  |
| 240 | TB0072 | Bone and joints | EPTB | SRR6257007 | Russia |  |
| 241 | TB0098 | Bone and joints | EPTB | SRR6257018 | Russia |  |
| 242 | 46-5069 | Central nervous system | EPTB | SRR5709738 | Thailand |  |
| 243 | Index 12-101 | Central nervous system | EPTB | SRR6480595 | Indonesia |  |
| 244 | Index 24-34 | Central nervous system | EPTB | SRR6480436 | Indonesia |  |
| 245 | Mtb-manila-36 | Lymph node | EPTB | SRR1510071 | Canada |  |
| 246 | DKC2-0492 | Lungs | Pulmonar | ERR2229367 | Denmark |  |
| 247 | Index 32-66 | Lungs | Pulmonar | SRR6480408 | Indonesia |  |
| 248 | Index 6-109 | Lungs | Pulmonar | SRR6480382 | Indonesia |  |
| 249 | Index 60-42 | Lungs | Pulmonar | SRR6480494 | Indonesia |  |
| 250 | Index 69-25 | Lungs | Pulmonar | SRR6480617 | Indonesia |  |
| 251 | DS-25878 | Lungs | Pulmonar | SRR5709875 | Thailand |  |
| 252 | c37_2008 | Lungs | Pulmonar | ERR1144995 | Australia |  |
| 253 | Index 37-133 | Lungs | Pulmonar | SRR6480326 | Indonesia |  |
| 254 | DS-27524 | Lungs | Pulmonar | SRR5709753 | Thailand |  |
| 255 | Index 73-100 | Lungs | Pulmonar | SRR6480601 | Indonesia |  |
| 256 | Mtb_ON-A_strain54 | Lungs | Pulmonar | SRR1573733 | Canada |  |
| 257 | DS-10110 | Lungs | Pulmonar | SRR5709802 | Thailand |  |
| 258 | DS-14191 | Lungs | Pulmonar | SRR5709823 | Thailand |  |
| 259 | DS-20257 | Lungs | Pulmonar | SRR5709943 | Thailand |  |
| 260 | DS-13949 | Lungs | Pulmonar | SRR5709820 | Thailand |  |
| 261 | DS-20915 | Lungs | Pulmonar | SRR5709770 | Thailand |  |
| 262 | c32_2012 | Lungs | Pulmonar | ERR1144994 | Australia |  |
| 263 | DS-11745 | Lungs | Pulmonar | SRR5709801 | Thailand |  |
| 264 | Index 42-21 | Lungs | Pulmonar | SRR6480573 | Indonesia |  |
| 265 | Index 42-100 | Lungs | Pulmonar | SRR6480343 | Indonesia |  |
| 266 | DS-19861 | Lungs | Pulmonar | SRR5709940 | Thailand |  |
| 267 | DS-13671 | Lungs | Pulmonar | SRR5709799 | Thailand |  |
| 268 | Index 15-25 | Lungs | Pulmonar | SRR6480545 | Indonesia |  |
| 269 | DS-18501 | Lungs | Pulmonar | SRR5709953 | Thailand |  |
| 270 | DS-16630 | Lungs | Pulmonar | SRR5709798 | Thailand |  |
| 271 | Index 28-142 | Lungs | Pulmonar | SRR6480411 | Indonesia |  |
| 272 | Index 21-106 | Lungs | Pulmonar | SRR6480509 | Indonesia |  |
| 273 | Index 66-109 | Lungs | Pulmonar | SRR6480345 | Indonesia |  |
| 274 | DCTB021 | Lungs | Pulmonar | SRR1002689 | Russia |  |
| 275 | Index 60-21 | Lungs | Pulmonar | SRR6480574 | Indonesia |  |
| 276 | DS-17016 | Lungs | Pulmonar | SRR5709892 | Thailand |  |
| 277 | RTS-3 | Lungs | Pulmonar | SRR6914120 | Russia |  |
| 278 | Index 15-100 | Lungs | Pulmonar | SRR6480401 | Indonesia |  |
| 279 | Index 32-90 | Lungs | Pulmonar | SRR6480318 | Indonesia |  |
| 280 | Index 55-109 | Lungs | Pulmonar | SRR6480628 | Indonesia |  |
| 281 | Index 12-100 | Lungs | Pulmonar | SRR6480402 | Indonesia |  |
| 282 | DS-14490 | Lungs | Pulmonar | SRR5709864 | Thailand |  |
| 283 | EKB43 | Lungs | Pulmonar | SRR8357275 | Russia |  |
| 284 | DS-20512 | Lungs | Pulmonar | SRR5710016 | Thailand |  |
| 285 | EKB25 | Lungs | Pulmonar | SRR8348943 | Russia |  |
| 286 | DS-33051 | Lungs | Pulmonar | SRR5709862 | Thailand |  |
| 287 | Index 40-133 | Lungs | Pulmonar | SRR6480328 | Indonesia |  |
| 288 | Index 12-106 | Lungs | Pulmonar | SRR6480512 | Indonesia |  |
| 289 | c30_2012 | Lungs | Pulmonar | ERR1144992 | Australia |  |
| 290 | EKB9 | Lungs | Pulmonar | SRR8335024 | Russia |  |
| 291 | DS-32353 | Lungs | Pulmonar | SRR5709911 | Thailand |  |
| 292 | Index 75-109 | Lungs | Pulmonar | SRR6480342 | Indonesia |  |
| 293 | Index 32-21 | Lungs | Pulmonar | SRR6480534 | Indonesia |  |
| 294 | Index 37-109 | Lungs | Pulmonar | SRR6480389 | Indonesia |  |
| 295 | EKB33 | Lungs | Pulmonar | SRR8439315 | Russia |  |
| 296 | RTS-6 | Lungs | Pulmonar | SRR6914123 | Russia |  |
| 297 | Index 24-90 | Lungs | Pulmonar | SRR6480634 | Indonesia |  |
| 298 | DS-16496 | Lungs | Pulmonar | SRR5709912 | Thailand |  |
| 299 | EKB83 | Lungs | Pulmonar | SRR8375802 | Russia |  |
| 300 | Index 6-21 | Lungs | Pulmonar | SRR6480576 | Indonesia |  |
| 301 | EKB24 | Lungs | Pulmonar | SRR8348431 | Russia |  |
| 302 | Index32-106 | Lungs | Pulmonar | SRR6480513 | Indonesia |  |
| 303 | Index 55-42 | Lungs | Pulmonar | SRR6480498 | Indonesia |  |
| 304 | EKB1 | Lungs | Pulmonar | SRR8327216 | Russia |  |
| 305 | Index 18-42 | Lungs | Pulmonar | SRR6480450 | Indonesia |  |
| 306 | DS-15504 | Lungs | Pulmonar | SRR5710009 | Thailand |  |
| 307 | DS-20092 | Lungs | Pulmonar | SRR5709961 | Thailand |  |
| 308 | JAL_IOB_199 | Lungs | Pulmonar | SRR5341273 | India |  |
| 309 | Index 45-142 | Lungs | Pulmonar | SRR6480499 | Indonesia |  |
| 310 | Index 6-106 | Lungs | Pulmonar | SRR6480421 | Indonesia |  |
| 311 | Index 45-66 | Lungs | Pulmonar | SRR6480446 | Indonesia |  |
| 312 | Index 18-25 | Lungs | Pulmonar | SRR6480546 | Indonesia |  |
| 313 | DS-30312 | Lungs | Pulmonar | SRR5710011 | Thailand |  |
| 314 | EKB7 | Lungs | Pulmonar | SRR8330159 | Russia |  |
| 315 | RTS-5 | Lungs | Pulmonar | SRR6914122 | Russia |  |
| 316 | DS-17092 | Lungs | Pulmonar | SRR5709889 | Thailand |  |
| 317 | DS-10471 | Lungs | Pulmonar | SRR5709948 | Thailand |  |
| 318 | c31_2012 | Lungs | Pulmonar | ERR1144993 | Australia |  |
| 319 | DS-29147 | Lungs | Pulmonar | SRR5709763 | Thailand |  |
| 320 | DS-17290 | Lungs | Pulmonar | SRR5709794 | Thailand |  |
| 321 | Index 73-142 | Lungs | Pulmonar | SRR6480358 | Indonesia |  |
| 322 | JAL_IOB_198 | Lungs | Pulmonar | SRR5341274 | India |  |
| 323 | Index 30-21 | Lungs | Pulmonar | SRR6480533 | Indonesia |  |
| 324 | Index 45-100 | Lungs | Pulmonar | SRR6480462 | Indonesia |  |
| 325 | Index 28-25 | Lungs | Pulmonar | SRR6480548 | Indonesia |  |
| 326 | DS-17984 | Lungs | Pulmonar | SRR5709888 | Thailand |  |
| 327 | DS-6882 | Lungs | Pulmonar | SRR5709933 | Thailand |  |
| 328 | Index 12-25 | Lungs | Pulmonar | SRR6480552 | Indonesia |  |
| 329 | EKB20 | Lungs | Pulmonar | SRR8346382 | Russia |  |
| 330 | DS-14607 | Lungs | Pulmonar | SRR5709954 | Thailand |  |
| 331 | DS-21363 | Lungs | Pulmonar | SRR5709991 | Thailand |  |
| 332 | Index 6-133 | Lungs | Pulmonar | SRR6480321 | Indonesia |  |
| 333 | Index 13-133 | Lungs | Pulmonar | SRR6480610 | Indonesia |  |
| 334 | EKB27 | Lungs | Pulmonar | SRR8352208 | Russia |  |
| 335 | RTS-2 | Lungs | Pulmonar | SRR6914119 | Russia |  |
| 336 | Index 42-66 | Lungs | Pulmonar | SRR6480447 | Indonesia |  |
| 337 | Index 40-42 | Lungs | Pulmonar | SRR6480539 | Indonesia |  |
| 338 | DS-19190 | Lungs | Pulmonar | SRR5709921 | Thailand |  |
| 339 | Index 73-21 | Lungs | Pulmonar | SRR6480567 | Indonesia |  |
| 340 | DS-15177 | Lungs | Pulmonar | SRR5709956 | Thailand |  |
| 341 | Index 78-106 | Lungs | Pulmonar | SRR6480431 | Indonesia |  |
| 342 | JAL_IOB_197 | Lungs | Pulmonar | SRR5341275 | India |  |
| 343 | Index 66-21 | Lungs | Pulmonar | SRR6480575 | Indonesia |  |
| 344 | Index 40-106 | Lungs | Pulmonar | SRR6480463 | Indonesia |  |
| 345 | DCTB018 | Lungs | Pulmonar | SRR1002684 | Russia |  |
| 346 | Index 30-34 | Lungs | Pulmonar | SRR6480438 | Indonesia |  |
| 347 | Index 61-66 | Lungs | Pulmonar | SRR6480440 | Indonesia |  |
| 348 | DS-19200 | Lungs | Pulmonar | SRR5709922 | Thailand |  |
| 349 | DS-12236 | Lungs | Pulmonar | SRR5709761 | Thailand |  |
| 350 | DKC2-0485 | Lungs | Pulmonar | ERR2229361 | Denmark |  |
| 351 | EKB36 | Lungs | Pulmonar | SRR8354461 | Russia |  |
| 352 | Index 6-142 | Lungs | Pulmonar | SRR6480495 | Indonesia |  |
| 353 | Index 40-109 | Lungs | Pulmonar | SRR6480390 | Indonesia |  |
| 354 | DS-16282 | Lungs | Pulmonar | SRR5709837 | Thailand |  |
| 355 | Index 34-100 | Lungs | Pulmonar | SRR6480336 | Indonesia |  |
| 356 | DS-23682 | Lungs | Pulmonar | SRR5709805 | Thailand |  |
| 357 | Index 55-142 | Lungs | Pulmonar | SRR6480497 | Indonesia |  |
| 358 | DS-20503 | Lungs | Pulmonar | SRR5709777 | Thailand |  |
| 359 | DS-31799 | Lungs | Pulmonar | SRR5709910 | Thailand |  |
| 360 | DS-23522 | Lungs | Pulmonar | SRR5709806 | Thailand |  |
| 361 | EKB28 | Lungs | Pulmonar | SRR8351921 | Russia |  |
| 362 | EKB38 | Lungs | Pulmonar | SRR8354716 | Russia |  |
| 363 | DS-31224 | Lungs | Pulmonar | SRR5709907 | Thailand |  |
| 364 | DS-20316 | Lungs | Pulmonar | SRR5709936 | Thailand |  |
| 365 | Index 42-109 | Lungs | Pulmonar | SRR6480391 | Indonesia |  |
| 366 | Index 69-42 | Lungs | Pulmonar | SRR6480359 | Indonesia |  |
| 367 | DS-31213 | Lungs | Pulmonar | SRR5710026 | Thailand |  |
| 368 | Index 37-142 | Lungs | Pulmonar | SRR6480544 | Indonesia |  |
| 369 | Index 42-142 | Lungs | Pulmonar | SRR6480507 | Indonesia |  |
| 370 | DKC2-0491 | Lungs | Pulmonar | ERR2229366 | Denmark |  |
| 371 | DS-29456 | Lungs | Pulmonar | SRR5709903 | Thailand |  |
| 372 | WT9 | Lungs | Pulmonar | SRR1573725 | Canada |  |
| 373 | Index 69-106 | Lungs | Pulmonar | SRR6480344 | Indonesia |  |
| 374 | EKB100 | Lungs | Pulmonar | SRR8434639 | Russia |  |
| 375 | DS-16280 | Lungs | Pulmonar | SRR5709946 | Thailand |  |
| 376 | Index 78-133 | Lungs | Pulmonar | SRR6480329 | Indonesia |  |
| 377 | WT17 | Lungs | Pulmonar | SRR1573730 | Canada |  |
| 378 | JAL_IOB_195 | Lungs | Pulmonar | SRR5341277 | India |  |
| 379 | Index 4-106 | Lungs | Pulmonar | SRR6480607 | Indonesia |  |
| 380 | Index 4_19 | Lungs | Pulmonar | SRR6480362 | Indonesia |  |
| 381 | Index 69-21 | Lungs | Pulmonar | SRR6480568 | Indonesia |  |
| 382 | DS-26102 | Lungs | Pulmonar | SRR5709749 | Thailand |  |
| 383 | WT16 | Lungs | Pulmonar | SRR1573729 | Canada |  |
| 384 | DS-17016 | Lungs | Pulmonar | SRR5709967 | Thailand |  |
| 385 | Index 45-133 | Lungs | Pulmonar | SRR6480327 | Indonesia |  |
| 386 | Index 37-106 | Lungs | Pulmonar | SRR6480518 | Indonesia |  |
| 387 | Index 21-21 | Lungs | Pulmonar | SRR6480530 | Indonesia |  |
| 388 | Index 75-106 | Lungs | Pulmonar | SRR6480428 | Indonesia |  |
| 389 | DS-19290 | Lungs | Pulmonar | SRR5709929 | Thailand |  |
| 390 | EKB15 | Lungs | Pulmonar | SRR8335313 | Russia |  |
| 391 | Index 37-90 | Lungs | Pulmonar | SRR6480316 | Indonesia |  |
| 392 | Index 55-133 | Lungs | Pulmonar | SRR6480322 | Indonesia |  |
| 393 | Index 40-101 | Lungs | Pulmonar | SRR6480563 | Indonesia |  |
| 394 | Index 55-100 | Lungs | Pulmonar | SRR6480399 | Indonesia |  |
| 395 | EKB19 | Lungs | Pulmonar | SRR8344409 | Russia |  |
| 396 | Index 75-100 | Lungs | Pulmonar | SRR6480604 | Indonesia |  |
| 397 | JAL_IOB_200 | Lungs | Pulmonar | SRR5341272 | India |  |
| 398 | Index 60-106 | Lungs | Pulmonar | SRR6480377 | Indonesia |  |
| 399 | DS-19383 | Lungs | Pulmonar | SRR5709962 | Thailand |  |
| 400 | Index 21-133 | Lungs | Pulmonar | SRR6480611 | Indonesia |  |
| 401 | DS-30442 | Lungs | Pulmonar | SRR5710012 | Thailand |  |
| 402 | 2184 | Lungs | Pulmonar | SRR8662666 | Russia |  |
| 403 | EKB18 | Lungs | Pulmonar | SRR8427096 | Russia |  |
| 404 | DS-21517 | Lungs | Pulmonar | SRR5709812 | Thailand |  |
| 405 | DKC2-0448 | Lungs | Pulmonar | ERR2229324 | Denmark |  |
| 406 | EKB42 | Lungs | Pulmonar | SRR8434867 | Russia |  |
| 407 | Index 24-21 | Lungs | Pulmonar | SRR6480535 | Indonesia |  |
| 408 | L2 | Lungs | Pulmonar | SRR6480540 | Indonesia |  |
| 409 | DS-18810 | Lungs | Pulmonar | SRR5709885 | Thailand |  |
| 410 | EKB99 | Lungs | Pulmonar | SRR8433589 | Russia |  |
| 411 | WT1 | Lungs | Pulmonar | SRR1573728 | Canada |  |
| 412 | Index 6-90 | Lungs | Pulmonar | SRR6480453 | Indonesia |  |
| 413 | DS-30951 | Lungs | Pulmonar | SRR5709881 | Thailand |  |
| 414 | EKB32 | Lungs | Pulmonar | SRR8353274 | Russia |  |
| 415 | Index 32-100 | Lungs | Pulmonar | SRR6480403 | Indonesia |  |
| 416 | Index12-21 | Lungs | Pulmonar | SRR6480531 | Indonesia |  |
| 417 | Index 12-109 | Lungs | Pulmonar | SRR6480502 | Indonesia |  |
| 418 | DS-10501 | Lungs | Pulmonar | SRR5710030 | Thailand |  |
| 419 | EKB65 | Lungs | Pulmonar | SRR8366603 | Russia |  |
| 420 | JAL_IOB_196 | Lungs | Pulmonar | SRR5341276 | India |  |
| 421 | Index 4-133 | Lungs | Pulmonar | SRR6480622 | Indonesia |  |
| 422 | EKB73 | Lungs | Pulmonar | SRR8369849 | Russia |  |
| 423 | Index 32-55 | Lungs | Pulmonar | SRR6480542 | Indonesia |  |
| 424 | Index15-42 | Lungs | Pulmonar | SRR6480458 | Indonesia |  |
| 425 | DS-25553 | Lungs | Pulmonar | SRR5709877 | Thailand |  |
| 426 | Index 32-133 | Lungs | Pulmonar | SRR6480621 | Indonesia |  |
| 427 | Index 12-90 | Lungs | Pulmonar | SRR6480520 | Indonesia |  |
| 428 | EKB17 | Lungs | Pulmonar | SRR8345980 | Russia |  |
| 429 | Index 78-21 | Lungs | Pulmonar | SRR6480550 | Indonesia |  |
| 430 | EKB31 | Lungs | Pulmonar | SRR8353479 | Russia |  |
| 431 | Index 24-106 | Lungs | Pulmonar | SRR6480516 | Indonesia |  |
| 432 | DS-32315 | Lungs | Pulmonar | SRR5709896 | Thailand |  |
| 433 | 4702 | Lungs | Pulmonar | SRR8662668 | Russia |  |
| 434 | DS-30056 | Lungs | Pulmonar | SRR5709960 | Thailand |  |
| 435 | DS-19155 | Lungs | Pulmonar | SRR5709927 | Thailand |  |
| 436 | EKB63 | Lungs | Pulmonar | SRR8366172 | Russia |  |
| 437 | Index 30-25 | Lungs | Pulmonar | SRR6480541 | Indonesia |  |
| 438 | EKB26 | Lungs | Pulmonar | SRR8351971 | Russia |  |
| 439 | DKC2-0446 | Lungs | Pulmonar | ERR2229322 | Denmark |  |
| 440 | Index 8-90 | Lungs | Pulmonar | SRR6480596 | Indonesia |  |
| 441 | EKB48 | Lungs | Pulmonar | SRR8358462 | Russia |  |
| 442 | DCTB003 | Lungs | Pulmonar | SRR993002 | Russia |  |
| 443 | WT15 | Lungs | Pulmonar | SRR1573727 | Canada |  |
| 444 | DCTB020 | Lungs | Pulmonar | SRR1002686 | Russia |  |
| 445 | DS-32512 | Lungs | Pulmonar | SRR5710028 | Thailand |  |
| 446 | Index 21-90 | Lungs | Pulmonar | SRR6480314 | Indonesia |  |
| 447 | DCTB001 | Lungs | Pulmonar | SRR974700 | Russia |  |
| 448 | Index 78-100 | Lungs | Pulmonar | SRR6480603 | Indonesia |  |
| 449 | Index 24-42 | Lungs | Pulmonar | SRR6480418 | Indonesia |  |
| 450 | EKB76 | Lungs | Pulmonar | SRR8370129 | Russia |  |
| 451 | DS-11221 | Lungs | Pulmonar | SRR5709796 | Thailand |  |
| 452 | Index 15-142 | Lungs | Pulmonar | SRR6480459 | Indonesia |  |
| 453 | BC14-Mtb515 | Lungs | Pulmonar | SRR6397999 | Canada |  |
| 454 | DCTB022 | Lungs | Pulmonar | SRR1002690 | Russia |  |
| 455 | EKB74 | Lungs | Pulmonar | SRR8369893 | Russia |  |
| 456 | DKC2-0483 | Lungs | Pulmonar | ERR2229359 | Denmark |  |
| 457 | DS-21644 | Lungs | Pulmonar | SRR5709928 | Thailand |  |
| 458 | DS-08775 | Lungs | Pulmonar | SRR5709793 | Thailand |  |
| 459 | Index 40-66 | Lungs | Pulmonar | SRR6480444 | Indonesia |  |
| 460 | Index 8-106 | Lungs | Pulmonar | SRR6480510 | Indonesia |  |
| 461 | EKB47 | Lungs | Pulmonar | SRR8358501 | Russia |  |
| 462 | DCTB014 | Lungs | Pulmonar | SRR1002679 | Russia |  |
| 463 | Index 73-42 | Lungs | Pulmonar | SRR6480357 | Indonesia |  |
| 464 | DS-20815 | Lungs | Pulmonar | SRR5709773 | Thailand |  |
| 465 | DS-6265 | Lungs | Pulmonar | SRR5709938 | Thailand |  |
| 466 | DKC2-0489 | Lungs | Pulmonar | ERR2229365 | Denmark |  |
| 467 | Index 75-21 | Lungs | Pulmonar | SRR6480549 | Indonesia |  |
| 468 | Index 69-100 | Lungs | Pulmonar | SRR6480602 | Indonesia |  |
| 469 | DS-20500 | Lungs | Pulmonar | SRR5709776 | Thailand |  |
| 470 | DS-29188 | Lungs | Pulmonar | SRR5709884 | Thailand |  |
| 471 | EKB22 | Lungs | Pulmonar | SRR8347539 | Russia |  |
| 472 | Index 28-42 | Lungs | Pulmonar | SRR6480412 | Indonesia |  |
| 473 | Index 18-21 | Lungs | Pulmonar | SRR6480529 | Indonesia |  |
| 474 | Index 75-90 | Lungs | Pulmonar | SRR6480420 | Indonesia |  |
| 475 | DS-15203 | Lungs | Pulmonar | SRR5709959 | Thailand |  |
| 476 | DS-19351 | Lungs | Pulmonar | SRR5709964 | Thailand |  |
| 477 | Index 60-66 | Lungs | Pulmonar | SRR6480441 | Indonesia |  |
| 478 | EKB21 | Lungs | Pulmonar | SRR8346061 | Russia |  |
| 479 | Index 4-90 | Lungs | Pulmonar | SRR6480315 | Indonesia |  |
| 480 | DS-18613 | Lungs | Pulmonar | SRR5709952 | Thailand |  |
| 481 | DS-11380 | Lungs | Pulmonar | SRR5709792 | Thailand |  |
| 482 | DS-17658 | Lungs | Pulmonar | SRR5709759 | Thailand |  |
| 483 | RTS-4 | Lungs | Pulmonar | SRR6914121 | Russia |  |
| 484 | EKB34 | Lungs | Pulmonar | SRR8439237 | Russia |  |
| 485 | DS-34062 | Lungs | Pulmonar | SRR5709860 | Thailand |  |
| 486 | DKC2-0487 | Lungs | Pulmonar | ERR2229363 | Denmark |  |
| 487 | Index 78-142 | Lungs | Pulmonar | SRR6480354 | Indonesia |  |
| 488 | DS-21639 | Lungs | Pulmonar | SRR5709813 | Thailand |  |
| 489 | Index 18-109 | Lungs | Pulmonar | SRR6480427 | Indonesia |  |
| 490 | Index 21-142 | Lungs | Pulmonar | SRR6480415 | Indonesia |  |
