# Supplementary material for: Whole-genome comparative analysis at the lineage/sublineage level discloses relationships between Mycobacterium tuberculosis genotype and clinical phenotype
Source: PeerJ. 2021 Sep 8;9:e12128. doi: 10.7717/peerj.12128 (PMC8434806; doi:10.7717/peerj.12128)
Supplement: Supplemental Information 2 — The frequencies of PTB / EPTB strains by lineage, sublineage and profile of resistance to antibiotics. These data were used for statistical analysis in order to search for possible associations. The results of this analysis are also shown in the table. [file peerj-09-12128-s002.docx]

**Supplemental table 2**. ﻿Associations between *Mycobacterium tuberculosis* lineage, sublineage and genotypic resistance with clinical phenotypes.

﻿

| Variable | | No. strains for: | | *P value^a^* | OR^a^  (95% CI) |  |
| --- | --- | --- | --- | --- | --- | --- |
|  |  | EPTB  *(n=245)* | PTB  *(n=245)* |  |  | Adjusted OR^b^ |
| Lineage | Lineage 1 | 38 | 9 | **0.000** | **4.8(2.2-10.1)** | **4.4(1.9-10.0)** |
|  | Lineage 2 | 112 | 154 | **0.002** | **1.5(1.1-2-2)** | **1.4 (1.1-1.9)** |
|  | Lineage 3 | 6 | 1 | 0.122 | 1.7(1.3-2.4) | 1.2 (0.7-1.8) |
|  | Lineage 4 | 89 | 81 | 0.448 | 0.8(0.8-1.1) | Reference |
| Sublineage | 1.1 | 1 | 0 | NA | NA | NA |
|  | 1.1.1 | 14 | 5 | **0.029** | **2.8(1.0-7.7)** | **2.9(1.0-7.9)** |
|  | 1.1.1.1 | 1 | 0 | NA | NA | NA |
|  | 1.1.2 | 1 | 0 | NA | NA | NA |
|  | 1.2.1 | 20 | 2 | **0.000** | **6.7(2.0-22.1)** | **7.2(1.6-31.7)** |
|  | 1.2.2 | 1 | 2 | NA | NA | NA |
|  | Asia Ancestral | 20 | 9 | **0.043** | **2.4(1.2-4.8)** | **2.3(1.2-5.0)** |
|  | Asian African 2 | 5 | 27 | **0.000** | **2.3(1.1-5.2)** | **2.7(2.2-7.8)** |
|  | Asian African 2 RD142 | 2 | 4 | NA | NA | NA |
|  | Asian African 3 | 21 | 15 | 0.060 | ﻿0.83 (0.41–1.69) | NA |
|  | Pacific RD150 | 11 | 14 | NA | NA | NA |
|  | Central Asia | 25 | 4 | **0.000** | **8.1(2.9-22.9)** | **9.4(2.9-28.4)** |
|  | Europe/Russia B0/W148 | 13 | 39 | **0.000** | **2.7(1.3-5.4)** | **3.1(1.7-6.0)** |
|  | Unclassified modern | 15 | 42 | **0.001** | **2.3(1.3-4)** | **3.0(1.7-5.9)** |
|  | 3 | 6 | 1 | 0.122 | 1.7(1.3-2.4) | NA |
|  | 4.1.1.3 | 1 | 0 | NA | NA | NA |
|  | 4.1.2 | 2 | 1 | NA | NA | NA |
|  | 4.1.2.1 | 19 | 3 | **0.000** | **6.3(1.9-21.1)** | **5.1(1.5-17.8)** |
|  | 4.2.1 | 4 | 0 | NA | NA | NA |
|  | 4.2.2 | 2 | 0 | NA | NA | NA |
|  | 4.3.1 | 7 | 18 | **0.031** | **2.9(1.2-7.7)** | **2.6(1.1-6.4)** |
|  | 4.3.2 | 3 | 0 | NA | NA | NA |
|  | 4.3.3 | 2 | 14 | **0.009** | **1.8(1.4-2.2)** | **1.7(1.6-2.4)** |
|  | 4.3.4.2 | 1 | 2 | NA | NA | NA |
|  | 4.4.1.1 | 4 | 0 | NA | NA | NA |
|  | 4.4.1.2 | 6 | 4 | NA | NA | NA |
|  | 4.4.2 | 6 | 4 | NA | NA | NA |
|  | 4.5 | 3 | 11 | **0.014** | **3.8(1.0-13.8)** | **4.8(1.4-16.7)** |
|  | 4.7 | 2 | 0 | NA | NA | NA |
|  | 4.8 | 27 | 24 | 0.325 | 0.6(0.6-1-2) | Reference |
| Genotypic resistance | Sensitive | 168 | 113 | **0.000** | **1.7(1.3-2-3)** | Reference |
|  | Drug resistant | 30 | 23 | 0.324 | 1.3(0.7-2.3) | NA |
|  | MDR | 41 | 75 | 0.657 | 1.1(0.7-1.6) | NA |
|  | XDR | 6 | 34 | **0.000** | **6.0(2.5-14.4)** | **6.0(2.3-14.4)** |

﻿Abbreviations: OR, odds ratio; CI, confidence interval; EPTB, extrapulmonary tuberculosis; PTB, pulmonary tuberculosis; MDR, multidrug-resistant tuberculosis; XDR, extensively drug-resistant tuberculosis; NA, not applicable.

**^a^** ﻿P value and odds ratio were calculated comparing the extrapulmonary and pulmonary proportions for each variable with Fisher’s exact test.

^b^ Adjusted ﻿ ORs with 95% confidence intervals including geographic region variable.

Bold value indicates a variable statistically significant at the level of 0.05
