# Supplementary material for: Whole-genome comparative analysis at the lineage/sublineage level discloses relationships between Mycobacterium tuberculosis genotype and clinical phenotype
Source: PeerJ. 2021 Sep 8;9:e12128. doi: 10.7717/peerj.12128 (PMC8434806; doi:10.7717/peerj.12128)
Supplement: Supplemental Information 3 — The frequency of the isolated strains of CNS, bone and joints, lymph, genitourinary system, lungs and extrapulmonary tuberculosis without defined anatomical isolation site. The frequencies are described in relation to lineages, sublineages and profiles of resistance to antibiotics and were used for statistical analysis. [file peerj-09-12128-s003.docx]

**Supplemental table 3**. ﻿Associations between *Mycobacterium tuberculosis* lineage, sublineage and genotypic resistance with anatomic region of isolation.

| Variable | | No. Strains for:  (P value; OR) | | Lymph | Genitourinary  system | EPTB | PTB |
| --- | --- | --- | --- | --- | --- | --- | --- |
|  |  | CNS | Bone and joints |  |  |  |  |
| Lineage | Lineage 1 | **31(0.000;3.9)** | 1 (NA) | 3 (NA) | 3 (NA) | 0 | 9 |
|  | Lineage 2 | 73 | **32 (0.001;1.5)** | 0 (NA) | 1 (NA) | 6(NA) | **154(0.002;1.5)** |
|  | Lineage 3 | 1(NA) | 0 (NA) | 5 (NA) | 0 (NA) | 0 | 1 |
|  | Lineage 4 | 72(.428;0.5) | 9 (NA) | 3 (NA) | 3 (NA) | 2 | 81 |
| Sublineage | 1.1 | 1 (NA) | 0 | 0 | 0 | 0 | 0 |
|  | 1.1.1 | **14 (0.045;2.8)** | 0 | 0 | 0 | 0 | 5 |
|  | 1.1.1.1 | 1 (NA) | 0 | 0 | 0 | 0 | 0 |
|  | 1.1.2 | 0 | 0 | 0 | 1 (NA) | 0 | 0 |
|  | 1.2.1 | **14 (0.009:6.7)** | 1 (NA) | 3 (NA) | 2 (NA) | 0 | 2 |
|  | 1.2.2 | 1 (NA) | 0 | 0 | 0 | 0 | 2 |
|  | Asia Ancestral | **20(0.043;2.4)** | 0 | 0 | 0 | 0 | 9 |
|  | Asian African 2 | 0 | 5 | 0 | 0 | 0 | **27(0.000;2.3)** |
|  | Asian African 2 RD142 | 2 (NA) | 0 | 0 | 0 | 0 | 4 |
|  | Asian African 3 | 15 (NA) | 6 (NA) | 0 | 0 | 0 | 15 |
|  | Pacific RD150 | 11 (NA) | 0 | 0 | 0 | 0 | 14 |
|  | Central Asia | **25(0.000;8.2)** | 0 | 0 | 0 | 0 | 4 |
|  | Europe/Russia B0/W148 | 0 | 13 | 0 | 0 | 0 | **39(0.000;2.7)** |
|  | Unclassified modern | 2 | 6 | 0 | 1 | 6 | **42(0.000)** |
|  | 3 | 1 (NA) | 0 | 5 (NA) | 0 | 0 | 1 (NA) |
|  | 4.1.1.3 | 1 (NA) | 0 | 0 | 0 | 0 | 0 |
|  | 4.1.2 | 2 (NA) | 0 | 0 | 0 | 0 | 1 (NA) |
|  | 4.1.2.1 | **15 (0.010;6.3)** | 4 | 0 | 0 | 0 | 3 |
|  | 4.2.1 | 0 | 2 (NA) | 0 | 0 | 2 (NA) | 0 |
|  | 4.2.2 | 0 | 1 (NA) | 1 (NA) | 0 | 0 | 0 |
|  | 4.3.1 | 7 | 0 | 0 | 0 | 0 | **18(0.031;2.9)** |
|  | 4.3.2 | 3 (NA) | 0 | 0 | 0 | 0 | 0 |
|  | 4.3.3 | 2 | 0 | 0 | 0 | 0 | **14(0.009;1.8)** |
|  | 4.3.4.2 | 1 (NA) | 0 | 0 | 0 | 0 | 2 |
|  | 4.4.1.1 | 4 (NA) | 0 | 0 | 0 | 0 | 0 |
|  | 4.4.1.2 | 6 (NA) | 0 | 0 | 0 | 0 | 4 |
|  | 4.4.2 | 6 (NA) | 0 | 0 | 0 | 0 | 4 |
|  | 4.5 | 3 | 0 | 0 | 0 | 0 | **11(0.014;3.8)** |
|  | 4.7 | 2 (NA) | 0 | 0 | 0 | 0 | 0 |
|  | 4.8 | 18 (NA) | 4 (NA) | 2 (NA) | 3 (NA) | 0 | 24 |
| Genotypic resistance | Sensitive | **144(0.024;1.4)** | 10 | 5 | 6 | 3 | 113 |
|  | Drug resistant | 26(0.660) | 4 | 0 | 0 | 0 | 23 |
|  | MDR | 7 | 23 | 6 | 1 | 4 | **75(0.000;1.9)** |
|  | XDR | 0 | 5 | 0 | 0 | 1 | **34(0.000;6.0)** |

Abbreviations: CNS, central nervous system; EPTB, extrapulmonary tuberculosis without classification; PTB, pulmonary tuberculosis; MDR, multidrug-resistant tuberculosis; XDR, extensively drug-resistant tuberculosis; NA, not applicable.

**^*^** ﻿P value was calculated comparing against pulmonary proportions for each variable with Fisher’s exact test. In PTB associations this was compared against CNS proportions.

Bold value indicates a variable statistically significant where p<0.05.
