# Supplementary material for: Whole-genome comparative analysis at the lineage/sublineage level discloses relationships between Mycobacterium tuberculosis genotype and clinical phenotype
Source: PeerJ. 2021 Sep 8;9:e12128. doi: 10.7717/peerj.12128 (PMC8434806; doi:10.7717/peerj.12128)
Supplement: Supplemental Information 4 — The spoligotypes identified in this study, as well as their frequency and the assigned family. These frequencies were used to search for associations between the spoligotype and the clinical phenotype of tuberculosis. [file peerj-09-12128-s004.docx]

| **Supplemental Table 4**. Identified families, spoligotypes and SITs. | | | | |
| --- | --- | --- | --- | --- |
| **Family ^b^** | **SIT ^c^** | **Binary code ^a^** | **Octal code ^a^** | **n (%)** |
| EAI2 | 19 | 1101111111111111111001111111000010111111111 | 677777477413771 | 13(2.65) |
|  | NR* | 0101111000000000000000000111000010111111111 | 274000003413771 | 1(0.2) |
|  | 89 | 1101111000000000000000000111000010111111111 | 674000003413771 | 9(1.8) |
| EAI3 | 11 | 1001111111111111111111111111000010110001111 | 477777777413071 | 1(0.2) |
| EAI4 | 139 | 1111111111111111111111111001000010111111111 | 777777774413771 | 1(0.2) |
|  | NR* | 1111111110111111011111111001000010111110011 | 777375774413711 | 1(0.2) |
| EAI5 | 349 | 1111111111110111111111111111000010111110111 | 777737777413731 | 1(0.2) |
|  | NR* | 1111111011111111111111111111000010011111111 | 775777777411771 | 1(0.2) |
|  | NR* | 1111111111111111110111111111000010111110000 | 777777377413700 | 1(0.2) |
|  | NR* | 1111111111110000001111111111000010001110000 | 777700777410700 | 1(0.2) |
|  | 256 | 1111111111111111111111111111000010111101111 | 777777777413671 | 5(1.02) |
|  | NR* | 0011111111111111111111111111000010111101111 | 177777777413671 | 1(0.2) |
|  | 951 | 1111111000011111111111111111000010111111111 | 774177777413771 | 2(0.4) |
|  | NR* | 1111111111111111000111111111000010111111111 | 777774377413771 | 1(0.2) |
|  | 204 | 1110111111111111111111111111000010111111111 | 737777777413771 | 1(0.2) |
|  | 947 | 1111111111111111111111111111000010111100111 | 777777777413631 | 1(0.2) |
|  | 735 | 1111111111111111101111111111000010111110111 | 777776777413731 | 1(0.2) |
|  | 939 | 1111111011111111111111111111000010111111111 | 775777777413771 | 1(0.2) |
|  | NR* | 1111111111110111111111111111000010111101111 | 777737777413671 | 1(0.2) |
|  | 517 | 1111111111111111111111111111000010111110011 | 777777777413711 | 1(0.2) |
|  | 236 | 1111111111111111111111111111000010111111111 | 777777777413771 | 1(0.2) |
|  | NR* | 1111111111111011111111111111000010111100111 | 777757777413631 | 1(0.2) |
| BEIJING | 190 | 0000000000000000000000000000000000111110111 | 000000000003731 | 2(0.4) |
|  | 1 | 0000000000000000000000000000000000111111111 | 000000000003771 | 224(45.71) |
|  | 269 | 0000000000000000000000000000000000001111111 | 000000000000771 | 22(4.48) |
|  | 621 | 0000000000000000000000000000000000101111111 | 000000000002771 | 1(0.2) |
|  | NR* | 0000000000000000000000000000000000100000000 | 000000000002000 | 13(2.65) |
|  | NR* | 0100000000000000000000000000000000111111111 | 200000000003771 | 1(0.2) |
| CAS | NR* | 1110000111111111011111000100000000111111111 | 703775742003771 | 1(0.2) |
|  | 356 | 1110000111111111111100000000000000011111111 | 703777600001771 | 1(0.2) |
|  | 26 | 1110000111111111111111000000000000111111111 | 703777740003771 | 1(0.2) |
|  | 25 | 1110000111111111111111000000000000110011111 | 703777740003171 | 1(0.2) |
|  | NR* | 1110000111111111011111000000000000111111111 | 703775740003771 | 3(0.6) |
| FAMILY33 | NR* | 1111111111111111111100111111111110111111111 | 777777637773771 | 1(0.2) |
|  | 523 | 1111111111111111111111111111111111111111111 | 777777777777771 | 1(0.2) |
| FAMILY34 | 46 | 1111111111111111111111110000000000000000000 | 777777770000000 | 1(0.2) |
|  | NR* | 1111101111111111111111000000000000000000000 | 767777740000000 | 1(0.2) |
|  | 402 | 1111111111111111111100000000000000000000000 | 777777600000000 | 1(0.2) |
| FAMILY35 | NR* | 1111111111101000000000000000000000000000000 | 777640000000000 | 1(0.2) |
| FAMILY36 | NR* | 0000000000000000000000001111000000000010000 | 000000007400100 | 1(0.2) |
|  | 4 | 0000000000000000000000001111111100001111111 | 000000007760771 | 3(0.6) |
| H37Rv-LIKE | NR* | 1111111111110011111001111111111100001111111 | 777717477760771 | 2(0.4) |
| HARLEEM1 | 620 | 1111111111111111111111001000000100001111111 | 777777744020771 | 1(0.2) |
|  | NR* | 1101111100111111111111100000000000000011111 | 676377760000171 | 1(0.2) |
|  | 218 | 1111111111110111111111111000000100001111111 | 777737774020771 | 1(0.2) |
|  | 316 | 1111111111111111111111110000000100001110111 | 777777770020731 | 3(0.6) |
|  | 742 | 1111111111111111111111110000000100001111111 | 777777770020771 | 3(0.6) |
|  | 47 | 1111111111111111111111111000000100001111111 | 777777774020771 | 1(0.2) |
|  | NR* | 1100111111111111111111110000000100001111111 | 637777770020771 | 1(0.2) |
|  | NR* | 1111111111111111111111000000000000000011111 | 777777740000171 | 1(0.2) |
|  | 1652 | 1001111111111111111111111000000100001111111 | 477777774020771 | 1(0.2) |
|  | NR* | 1111111111111111110001110000000100001111111 | 777777070020771 | 1(0.2) |
| HARLEEM3 | 777 | 1111111111111111111111111111000100001111111 | 777777777420771 | 2(0.4) |
|  | 1229 | 1111111111111000111111111111110100001111111 | 777743777720771 | 1(0.2) |
|  | NR* | 1111111111110111111011111111000100001110111 | 777737577420731 | 1(0.2) |
|  | 262 | 1111111001111111111111111111000100001111111 | 774777777420771 | 2(0.4) |
|  | NR* | 0000111110011111111111111111110100001111111 | 037177777720771 | 1(0.2) |
|  | 49 | 1111111111111111111111111111110100001110111 | 777777777720731 | 3(0.6) |
| LAM1 | 469 | 0001111111111111111100001111111100001111111 | 077777607760771 | 1(0.2) |
| LAM3 | 33 | 1111111100011111111100001111111100001111111 | 776177607760771 | 3(0.6) |
|  | NR | 1111000000000001111100001101111100001111111 | 740007606760771 | 1(0.2) |
| LAM7 | 32 | 1111111100000000000000000000000000000011111 | 776000000000171 | 2(0.4) |
| LAM8 | NR* | 1111111111111111111100000000000000001110011 | 777777600000711 | 1(0.2) |
|  | NR* | 1111000001111111111100000000000000000111111 | 740777600000371 | 3(0.6) |
|  | NR | 1111000001110001111100000000000000000111111 | 740707600000371 | 1(0.2) |
|  | NR* | 1111111111111111111100000000000000000001111 | 777777600000071 | 1(0.2) |
| LAM9 | NR* | 0111111111111111111100001111100100001111111 | 377777607620771 | 1(0.2) |
|  | 42 | 1111111111111111111100001111111100001111111 | 777777607760771 | 4(0.8) |
|  | 1277 | 1111111111111111110100001111111100001111111 | 777777207760771 | 1(0.2) |
|  | 1709 | 1111111111110111111100001101111100001111111 | 777737606760771 | 2(0.4) |
|  | 290 | 1111111111111111111100001101111100001111111 | 777777606760771 | 6(1.2) |
|  | 388 | 1110111111111111111100001111111100001111111 | 737777607760771 | 2(0.4) |
|  | 398 | 1111111111111111111100001111111100001100111 | 777777607760631 | 1(0.2) |
|  | 1708 | 1111111101111111111100001111111100001111111 | 776777607760771 | 1(0.2) |
|  | NR* | 0111111111111111111100001111100100001111011 | 377777607620751 | 1(0.2) |
|  | 770 | 1111111111101111111100001111111100001111111 | 777677607760771 | 1(0.2) |
|  | NR* | 1111111111111111111100001101111100001110011 | 777777606760711 | 1(0.2) |
|  | NR* | 1111111111111111110100001111111100001110111 | 777777207760731 | 1(0.2) |
|  | 452 | 1111111111111111111100001100111100001111111 | 777777606360771 | 1(0.2) |
|  | NR* | 1111111111100011111100001101111100001111111 | 777617606760771 | 1(0.2) |
| S | 34 | 1111111100111111111111111111111100001111111 | 776377777760771 | 1(0.2) |
| T1 | NR* | 1000001111110000111111111111111100001111111 | 407703777760771 | 1(0.2) |
|  | 334 | 1011111111111111111111111111111100001111111 | 577777777760771 | 1(0.2) |
|  | 196 | 1101111111111111111111111111111100001111111 | 677777777760771 | 1(0.2) |
|  | NR* | 1111111111110000111111111111111100001110111 | 777703777760731 | 2(0.4) |
|  | 44 | 1111111111111111111111011111111100001111111 | 777777757760771 | 1(0.2) |
|  | NR* | 1110111111110000111111111111111100001111111 | 737703777760771 | 1(0.2) |
|  | 370 | 1111111111111111111111001111111100001001111 | 777777747760471 | 2(0.4) |
|  | 120 | 1111111111111111111011111111111100001111111 | 777777577760771 | 1(0.2) |
|  | 358 | 1110011111111111111111111111111100001111111 | 717777777760771 | 1(0.2) |
|  | 917 | 1111111111011111111111111111111100001111111 | 777577777760771 | 1(0.2) |
|  | 53 | 1111111111111111111111111111111100001111111 | 777777777760771 | 39(7.9) |
|  | 804 | 1001111111111111111111111111111100001111111 | 477777777760771 | 6(1.2) |
|  | 52 | 1111111111111111111111111111111100001110111 | 777777777760731 | 5(1.02) |
|  | 1122 | 1111101111111111111111111111111100001111111 | 767777777760771 | 6(1.2) |
|  | 102 | 1111111111110000111111111111111100001111111 | 777703777760771 | 5(1.02) |
|  | NR* | 1011111111111111111011111111111100001111111 | 577777577760771 | 1(0.2) |
|  | NR* | 0010111111111111111101111111111100001111111 | 137777677760771 | 1(0.2) |
|  | NR* | 1111111111110111111011111111111100001111111 | 777737577760771 | 1(0.2) |
|  | NR* | 1001111111110111111111111111111100001111111 | 477737777760771 | 1(0.2) |
|  | 291 | 1111111111111111111101111111111100001111111 | 777777677760771 | 1(0.2) |
|  | 1655 | 1111111111110100111111111111111100001111111 | 777723777760771 | 1(0.2) |
|  | 253 | 1111111111111111111101100111111100001111111 | 777777663760771 | 1(0.2) |
|  | NR* | 1110111111111111111101111101111100001111111 | 737777676760771 | 1(0.2) |
|  | NR* | 1111111111110000111111111111111100001110011 | 777703777760711 | 1(0.2) |
|  | NR* | 1100000011110011111111111111111100001111111 | 601717777760771 | 1(0.2) |
|  | 37 | 1111111111110111111111111111111100001111111 | 777737777760771 | 2(0.4) |
|  | 498 | 1111111111101111111111111111111100001111111 | 777677777760771 | 1(0.2) |
|  | NR* | 1111111110000111111111111111111100001110011 | 777037777760711 | 1(0.2) |
|  | 175 | 1111111111111111111101111111111100001110111 | 777777677760731 | 1(0.2) |
| T2 | NR* | 1111111111111111111111111111111000000000000 | 777777777740000 | 1(0.2) |
| T3 | 1491 | 1111000000000000000000001111111100001110111 | 740000007760731 | 5(1.02) |
|  | NR* | 1111000000000000001111111111111100001111111 | 740000777760771 | 1(0.2) |
| T4 | 249 | 1111111111101000000000001111111100001111111 | 777640007760771 | 1(0.2) |
|  | 567 | 1111111111111100000000001111111100111111111 | 777760007763771 | 1(0.2) |
| X1 | NR* | 1111111111110110101100011111111100001111111 | 777732617760771 | 1(0.2) |
| X3 | 1751 | 1110000000001101101111111111111100001111111 | 700066777760771 | 1(0.2) |
| ^a^ Identified using Spotyping  ^b^ Family derived using TB-insight  ^c^ Identified using the SITVIT database  SIT: Shared international type. n: number of isolates  NR* Spoligotype not reported in SITVIT database | | | | |
