# Supplementary material for: Whole-genome comparative analysis at the lineage/sublineage level discloses relationships between Mycobacterium tuberculosis genotype and clinical phenotype
Source: PeerJ. 2021 Sep 8;9:e12128. doi: 10.7717/peerj.12128 (PMC8434806; doi:10.7717/peerj.12128)
Supplement: Supplemental Information 5 — The mutations associated with antibiotic resistance and the frequency observed. These data were used to search for associations between specific mutations and the extrapulmonary phenotype, and to define the assigned resistance profiles. [file peerj-09-12128-s005.docx]

| **Supplemental Table 5.** Identified mutations in genes that are known to confer resistance to first and second-line drugs. | | | | | | |
| --- | --- | --- | --- | --- | --- | --- |
| **Drug / resistance gene** | **Nucleotide position** | **Codon position** | **Polimorfism** | **Aminoacid change** | **Number of isolates PTB** | **Number of isolates EPTB** |
| RIF/*rpo*B | 508 | 170 | GTC/TTC | Val/Phe | 1 | 1 |
| RIF/*rpo*B | 1198 | 400 | ACC/GCC | Thr/Ala | 1 | 0 |
| RIF/rpoB | 1289 | 430 | CTG/CCG | Leu/Pro | 0 | 2 |
| RIF/*rpo*B | 1288 | 430 | CTG/GTG | Leu/Val | 1 | 0 |
| RIF/*rpo*B | 1295 | 432 | CAA/CTA | Gln/Leu | 1 | 0 |
| RIF/*rpo*B | 1297 | 433 | insTTC | Ins Phe | 2 | 1 |
| RIF/*rpo*B | 1303 | 435 | GAC/TAC | Asp/Tyr | 6 | 3 |
| RIF/*rpo*B | 1304 | 435 | GAC/GTC | Asp/Val | 8 | 1 |
| RIF/*rpo*B | 1304 | 435 | GAC/GGC | Asp/Gly | 1 | 0 |
| RIF/*rpo*B | 1322 | 441 | TCG/TTG | Ser/Leu | 1 | 1 |
| RIF/*rpo*B | 1334 | 445 | CAC/CGC | His/Arg | 2 | 1 |
| RIF/*rpo*B | 1333 | 445 | CAC/GAC | His/Asp | 5 | 1 |
| RIF/*rpo*B | 1333 | 445 | CAC/AAC | His/Asn | 1 | 0 |
| RIF/*rpo*B | 1333 | 445 | CAC/TAC | His/Tyr | 8 | 1 |
| RIF/*rpo*B | 1334 | 445 | CAC/CTC | His/Leu | 5 | 0 |
| RIF/*rpo*B | 1334 | 445 | CAC/CGC | His/Leu | 1 | 0 |
| RIF/*rpo*B | 1349 | 450 | TCG/TGG | Ser/Trp | 1 | 0 |
| RIF/*rpo*B | 1349 | 450 | TCG/TTG | Ser/Leu | 65 | 21 |
| RIF/*rpo*B | 1355 | 452 | CTG/CCG | Leu/Pro | 3 | 3 |
| RIF/*rpo*B | 1379 | 460 | GAG/GGG | Glu/Gly | 3 | 0 |
| RIF/*rpo*B | 1390 | 464 | CTG/ATG | Leu/Met | 0 | 1 |
| RIF/*rpo*B | 1396 | 466 | AAA/AAG | Lys/Gln | 1 | 0 |
| RIF/*rpo*B | 2283 | 761 | GAG/GAT | Glu/Asp | 0 | 6 |
| RIF/*rpoC* | 1472 | 491 | ATC/ACC | Ile/Thr | 8 | 2 |
| RIF/*rpoC* | 1579 | 527 | CTG/GTG | Leu/Val | 2 | 1 |
| PAS/*fol*C | 119 | 40 | GAG/GGG | Glu/Gly | 10 | 0 |
| PAS/*fol*C | 128 | 43 | ATC/ACC | Ile/Thr | 4 | 0 |
| PAS/*fol*C | 145 | 49 | CGG/TGG | Arg/Trp | 1 | 1 |
| PAS/*fol*C | 448 | 150 | AGC/GGC | Ser/Gly | 6 | 2 |
| PAS/*fol*C | 458 | 153 | GAG/GCG | Glu/Ala | 1 | 1 |
| PAS/*thyA* | 64 | 22 | ACC/GCC | Thr/Ala | 2 | 0 |
| PAS/*thyA* | 249 | 83 | TAT/TAG | Trp/STOP | 1 | 0 |
| PAS/*thyX* | -16 | — | C/T | — | 0 | 2 |
| PAS/*thyX* | 119 | 40 | GAG/GGG | Glu/Gly | 10 | 0 |
| PAS/*thyX* | 128 | 43 | ATC/ACC | Ile/Thr | 4 | 0 |
| AMI/*rrs* | 1401 | — | A/G | — | 23 | 6 |
| AMI/*rrs* | 1402 | — | C/A | — | 1 | 0 |
| AMI/*rrs* | 1484 | — | G/T | — | 1 | 0 |
| FLQ/*gyr*A | 269 | 90 | GTT/GCT | Ala/Val | 14 | 0 |
| FLQ/*gyr*A | 271 | 91 | TCG/CCG | Ser/Pro | 4 | 1 |
| FLQ/*gyr*A | 281 | 94 | GAC/GTC | Asp/Val | 1 | 0 |
| FLQ/*gyr*A | 280 | 94 | GAC/CAC | Asp/His | 2 | 0 |
| FLQ/*gyr*A | 280 | 94 | GAC/TAC | Asp/tyr | 5 | 0 |
| FLQ/*gyr*A | 281 | 94 | GAC/GCC | Asp/Ala | 2 | 0 |
| FLQ/*gyr*A | 280 | 94 | GAC/AAC | Asp/Asn | 3 | 2 |
| FLQ/*gyr*A | 281 | 94 | GAC/GGC | Asp/Gly | 24 | 6 |
| SM/*gid* | 102 | 34 | del 1 bp | Frameshift | 2 | 1 |
| SM/*gid* | 351 | 117 | del 1 bp | Frameshift | 0 | 1 |
| SM/*rps*L | 128 | 43 | AAG/AGG | Lys/Arg | 83 | 30 |
| SM/*rps*L | 263 | 88 | AAG/AGG | Lys/Arg | 7 | 7 |
| SM/*rps*L | 263 | 88 | AAG/ACG | Lys/Thr | 0 | 1 |
| SM/*rrs* | 514 | — | A/C | — | 3 | 0 |
| SM/*rrs* | 517 | — | C/T | — | 0 | 12 |
| SM/*rrs* | 888 | — | G/A | — | 0 | 1 |
| SM/*rrs* | 907 | — | A/C | — | 0 | 3 |
| SM/*rrs* | 907 | — | A/T | — | 0 | 1 |
| EMB/*emb*A | -11 | — | C/A | — | 2 | 1 |
| EMB/*emb*A | -12 | — | C/T | — | 5 | 0 |
| EMB/*emb*A | -16 | — | C/G | — | 0 | 1 |
| EMB/*emb*A | -16 | — | C/T | — | 4 | 0 |
| EMB/*emb*B | 886 | 296 | AAT/CAT | Asn/His | 4 | 0 |
| EMB/*emb*B | 916 | 306 | ATG/ATA | Met/Ile | 15 | 2 |
| EMB/*emb*B | 916 | 306 | ATG/GTG | Met/Val | 29 | 18 |
| EMB/*emb*B | 916 | 306 | ATG/CTG | Met/Leu | 0 | 1 |
| EMB/*emb*B | 956 | 319 | TAT/TCT | Tyr/Ser | 5 | 0 |
| EMB/*emb*B | 982 | 328 | GAC/TAC | Asp/Tyr | 3 | 0 |
| EMB/*emb*B | 1040 | 347 | AGC/ATC | Ser/Ile | 0 | 1 |
| EMB/*emb*B | 1061 | 354 | GAC/GCC | Asp/Ala | 4 | 3 |
| EMB/*emb*B | 1067 | 356 | GCG/GTG | Ala/Val | 0 | 1 |
| EMB/*emb*B | 1217 | 406 | GGC/AGC | Gly/Ser | 5 | 0 |
| EMB/*emb*B | 1217 | 406 | GGC/GAC | Gly/Asp | 5 | 3 |
| EMB/*emb*B | 1217 | 406 | GGC/GCC | Gly/Ala | 3 | 2 |
| EMB/*emb*B | 1490 | 497 | CAG/CCG | Gln/Arg | 15 | 1 |
| EMB/*emb*B | 1490 | 497 | CAG/CCG | Gln/Pro | 1 | 0 |
| EMB/*emb*B | 1490 | 497 | CAG/AAG | Gln/Lys | 5 | 0 |
| ETH/*eth*A | 11 | 4 | del 1bp | Frameshift | 0 | 1 |
| ETH/*eth*A | 110 | 36 | del 1bp | Frameshift | 10 | 3 |
| ETH/*eth*A | 180 | 60 | del 1bp | Frameshift | 0 | 1 |
| ETH/*eth*A | 341 | 114 | del 1bp | Frameshift | 0 | 3 |
| ETH/*eth*A | 509 | 169 | Del 1bp | Frameshift | 1 | 0 |
| ETH/*eth*A | 482 | 169 | del 2 bp | Frameshift | 1 | 0 |
| ETH/*eth*A | 639 | 213 | del 2bp | Frameshift | 17 | 3 |
| ETH/*eth*A | 640 | 214 | del 2bp | Frameshift | 0 | 1 |
| ETH/*eth*A | 703 | 234 | del 1bp | Frameshift | 0 | 1 |
| ETH/*eth*A | 768 | 256 | del 1bp | Frameshift | 0 | 2 |
| ETH/*eth*A | 771 | 257 | del 1bp | Frameshift | 1 | 0 |
| ETH/*eth*A | 805 | 269 | CAG/TAG | Gln/STOP | 0 | 1 |
| ETH/*eth*A | 851 | 283 | del 1bp | Frameshift | 1 | 0 |
| ETH/*eth*A | 1010 | 337 | del 1bp | Frameshift | 12 | 3 |
| ETH/*eth*A | 1011 | 337 | del 1bp | Frameshift | 2 | 1 |
| ETH/*eth*A | 1025 | 342 | ACA/AAA | Thr/Lys | 1 | 0 |
| ETH/*eth*A | 1054 | 352 | del 1bp | Frameshift | 1 | 0 |
| ETH/*eth*A | 1120 | 374 | del 2bp | Frameshift | 0 | 2 |
| ETH/*eth*A | 1154 | 385 | GGT/GAT | Gly/Asp | 0 | 1 |
| ETH/*eth*A | 1242 | 414 | insT | Frameshift | 0 | 1 |
| ETH/*eth*A | 1290 | 430 | del 1bp | Frameshift | 1 | 0 |
| ETH/*eth*A | 1307 | 435 | insC | Frameshift | 1 | 0 |
| ETH/*eth*A | 1343 | 448 | del 1bp | Frameshift | 0 | 1 |
| ETH/*eth*A | 1392 | 464 | insC | Frameshift | 1 | 0 |
| INH/*fab*G1 | -8 | — | T/C | — | 1 | 0 |
| INH/*fab*G1 | -15 | — | C/T | — | 21 | 9 |
| INH/*fab*G1 | -17 | — | G/T | — | 1 | 0 |
| INH/*inh*A | 118 | 40 | GGG/TGG | Gly/Trp | 1 | 0 |
| INH/*inh*A | 280 | 94 | TCT/GCT | Ser/Ala | 0 | 2 |
| INH/*ahpC* | -48 | — | G/A | — | 2 | 0 |
| INH/*ahpC* | -74 | — | G/A | — | 2 | 0 |
| INH/*ahpC* | -81 | — | C/T | — | 1 | 0 |
| INH/*kat*G | 589 | 197 | ins GG | Frameshift | 0 | 1 |
| INH/*kat*G | 771 | 257 | ATG/ATT | Met/Ile | 1 | 0 |
| INH/*kat*G | 944 | 315 | AGC/ACC | Ser/Thr | 101 | 69 |
| INH/*kat*G | 1054 | 352 | CAA/TAA | Gln/STOP | 1 | 0 |
| AMI/*rrs* | 1401 | — | A/T | — | 17 | 6 |
| AMI/*rrs* | 1402 | — | C/A | — | 1 | 0 |
| AMI/*rrs* | 1484 | — | G/T | — | 1 | 0 |
| CS/*alr* | 338 | 113 | CTC/CGC | Leu/Arg | 1 | 0 |
| PZA/*pnc*A | -11 | — | A/C | — | 0 | 1 |
| PZA/*pnc*A | -11 | — | A/G | Frameshift | 3 | 1 |
| PZA/*pnc*A | 50 | 17 | insG | Frameshift | 1 | 0 |
| PZA/*pnc*A | 52 | 17 | del 1 bp | Frameshift | 1 | 0 |
| PZA/*pnc*A | 98 | 33 | insG | Frameshift | 1 | 0 |
| PZA/*pnc*A | 139 | 46 | del 1 bp | Frameshift | 2 | 1 |
| PZA/*pnc*A | 250 | 83 | del 2 bp | Frameshift | 0 | 1 |
| PZA/*pnc*A | 279 | 93 | del 1bp | Frameshift | 1 | 0 |
| PZA/*pnc*A | 290 | 96 | del 1 bp | Frameshift | 1 | 0 |
| PZA/*pnc*A | 391 | 130 | insG | Frameshift | 1 | 0 |
| PZA/*pnc*A | 407 | 135 | insA | Frameshift | 1 | 0 |
| PZA/*pnc*A | 457 | 152 | insA | Frameshift | 1 | 0 |
| PZA/*pnc*A | 475 | 158 | del 1 bp | Frameshift | 0 | 1 |
| PZA/*pnc*A | 520 | 173 | insGG | Frameshift | 1 | 0 |
| PZA/*pnc*A | 524 | 174 | del 1 bp | Frameshift | 1 | 0 |
| PZA/*pnc*A | 527 | 175 | insG | Frameshift | 1 | 0 |
| PZA/*pnc*A | 534 | 178 | del 1 bp | Frameshift | 1 | 0 |
| PZA/*pnc*A | 309 | 103 | TAC/TAG | Tyr/STOP | 1 | 3 |
| PZA/*pnc*A | 8 | 3 | GCG/GAG | Ala/Glu | 0 | 2 |
| PZA/*pnc*A | 11 | 4 | TTG/TGG | Leu/Trp | 1 | 0 |
| PZA/*pnc*A | 14 | 5 | ATC/AGC | Ile/Ser | 1 | 0 |
| PZA/*pnc*A | 14 | 5 | ATC/ACC | Ile/Thr | 1 | 0 |
| PZA/*pnc*A | 23 | 8 | GAC/GCC | Asp/Ala | 1 | 0 |
| PZA/*pnc*A | 26 | 9 | GTA/GCA | Val/Ala | 1 | 1 |
| PZA/*pnc*A | 28 | 10 | CAG/AAG | Gln/Lys | 1 | 0 |
| PZA/*pnc*A | 29 | 10 | CAG/CCG | Gln/Pro | 0 | 1 |
| PZA/*pnc*A | 35 | 12 | GAT/GCT | Asp/Ala | 1 | 0 |
| PZA/*pnc*A | 41 | 14 | TGT/TAT | Cys/Tyr | 2 | 0 |
| PZA/*pnc*A | 52 | 18 | GGG/CGG | Gly/Arg | 1 | 0 |
| PZA/*pnc*A | 80 | 27 | CTA/CCA | Leu/Pro | 1 | 0 |
| PZA/*pnc*A | 92 | 31 | ATT/AGT | Ile/Ser | 0 | 1 |
| PZA/*pnc*A | 131 | 44 | GTA/GGA | Val/Gly | 1 | 0 |
| PZA/*pnc*A | 139 | 47 | ACA/CCA | Thr/Pro | 1 | 0 |
| PZA/*pnc*A | 146 | 49 | GAT/GGT | Asp/Gly | 1 | 0 |
| PZA/*pnc*A | 151 | 51 | CAC/GAC | His/Asp | 0 | 1 |
| PZA/*pnc*A | 161 | 54 | CCC/CTC | Pro/Leu | 0 | 1 |
| PZA/*pnc*A | 169 | 57 | CAT/GAT | His/Asp | 1 | 0 |
| PZA/*pnc*A | 172 | 58 | TTT/CTT | Phe/Leu | 0 | 1 |
| PZA/*pnc*A | 175 | 59 | TCA/CCA | Ser/Pro | 1 | 0 |
| PZA/*pnc*A | 185 | 62 | CCA/CTA | Pro/Leu | 1 | 0 |
| PZA/*pnc*A | 188 | 63 | GAT/GCT | Asp/Ala | 2 | 0 |
| PZA/*pnc*A | 199 | 67 | TCG/CCG | Ser/Pro | 1 | 0 |
| PZA/*pnc*A | 204 | 68 | TGG/TGC | Trp/Cys | 2 | 0 |
| PZA/*pnc*A | 204 | 68 | TGG/TGA | Trp/STOP | 1 | 0 |
| PZA/*pnc*A | 214 | 72 | CGC/TGC | Cys/Arg | 0 | 1 |
| PZA/*pnc*A | 228 | 76 | ACT/ATT | Thr/Ile | 1 | 0 |
| PZA/*pnc*A | 245 | 82 | CAC/CGC | His/Arg | 4 | 1 |
| PZA/*pnc*A | 289 | 90 | ATT/AGT | Ile/Ser | 1 | 0 |
| PZA/*pnc*A | 281 | 94 | TTT/TCT | Phe/Ser | 1 | 0 |
| PZA/*pnc*A | 286 | 96 | AAA/CAA | Lys/Gln | 0 | 1 |
| PZA/*pnc*A | 289 | 97 | GGT/AGT | Gly/Ser | 2 | 0 |
| PZA/*pnc*A | 290 | 97 | GGT/GAT | Gly/Asp | 0 | 1 |
| PZA/*pnc*A | 309 | 103 | TAT/TAA | Tyr/STOP | 0 | 0 |
| PZA/*pnc*A | 310 | 104 | AGT/CGT | Ser/Arg | 1 | 0 |
| PZA/*pnc*A | 357 | 119 | TGG/TGC | Trp/Cys | 5 | 0 |
| PZA/*pnc*A | 359 | 120 | CTT/CCT | Leu/Pro | 1 | 1 |
| PZA/*pnc*A | 403 | 135 | ACT/CCT | Thr/Pro | 0 | 1 |
| PZA/*pnc*A | 406 | 136 | GAT/GGT | Asp/Gly | 1 | 0 |
| PZA/*pnc*A | 412 | 138 | TGT/CGT | Cys/Arg | 0 | 1 |
| PZA/*pnc*A | 416 | 139 | GTT/GCT | Val/Ala | 1 | 1 |
| PZA/*pnc*A | 416 | 139 | GTT/GGT | Val/Gly | 1 | 1 |
| PZA/*pnc*A | 424 | 142 | ACT/GCT | Thr/Ala | 1 | 0 |
| PZA/*pnc*A | 478 | 160 | ACC/CCC | Thr/Pro | 0 | 1 |
| PZA/*pnc*A | 490 | 164 | TCG/CCG | Ser/Pro | 3 | 0 |
| PZA/*pnc*A | 512 | 171 | GCT/GTT | Ala/Val | 1 | 0 |
| Abbreviations: RIF, Rifampicin; PAS, Para-aminosalisylic acid; AMI, Aminoglycosides; FLQ, Fluoroquinolones; SM, Streptomycin; EMB, Ethambutol; ETH, Ethionamide; INH, Isoniazid; CS, Cicloserine; PZA, Pyrazinamide | | | | | | |
