# Supplementary material for: Whole-genome comparative analysis at the lineage/sublineage level discloses relationships between Mycobacterium tuberculosis genotype and clinical phenotype
Source: PeerJ. 2021 Sep 8;9:e12128. doi: 10.7717/peerj.12128 (PMC8434806; doi:10.7717/peerj.12128)
Supplement: Supplemental Information 6 — The frequency of strains isolated from CNS, bone and joints, lymph, genitourinary system, lungs and extrapulmonary tuberculosis without defined anatomical isolation site that presented genotypic resistance to first- and second-line antibiotics are shown in Table S6, these data were subjected to a statistical analysis to determine associations between the anatomical site of isolation and resistance to a given antibiotic. [file peerj-09-12128-s006.docx]

| **Supplemental Table 6.** Frequency of extrapulmonary resistant strains by antibiotic. | | | | | | |
| --- | --- | --- | --- | --- | --- | --- |
| **Drug** | *Number of resistant strains:* | | | | | |
|  | **Central nervous system** | **Bone and joints** | **Lymph** | **Genitourinary system** | **EPTB** | **PTB** |
| Isoniazid | 24 | 32 | 1 | 6 | 5 | 122 |
| Rifampicin | 8 | 28 | 1 | 6 | 5 | 116 |
| Pyrazinamide | 3 | 18 | 1 | 0 | 4 | 74 |
| Streptomycin | 13 | 31 | 0 | 2 | 5 | 101 |
| Ethambutol | 7 | 23 | 1 | 4 | 4 | 94 |
| Ethionamide | 12 | 18 | 0 | 0 | 2 | 65 |
| Flouroquinolones | 3 | 6 | 0 | 0 | 2 | 65 |
| Aminoglycosides | 0 | 4 | 0 | 0 | 2 | 18 |
| Para-aminosalisylic acid | 2 | 4 | 0 | 0 | 0 | 30 |
| Cicloserine | 0 | 0 | 0 | 0 | 0 | 1 |
| Abbreviations: EPTB, extrapulmonary tuberculosis without classification; PTB, pulmonary tuberculosis | | | | | | |
